# Discrepancies between Patients’ Preferences and Educational Programs on Oral Anticoagulant Therapy: A Survey in Community Pharmacies and Hospital Consultations

**DOI:** 10.1371/journal.pone.0146927

**Published:** 2016-01-14

**Authors:** Diane Macquart de Terline, Gilles Hejblum, Christine Fernandez, Ariel Cohen, Marie Antignac

**Affiliations:** 1 AP–HP, Hôpital Saint-Antoine, Service de Pharmacie, Paris, France; 2 Sorbonne Universités, UPMC Univ Paris 06, INSERM, Institut Pierre Louis d’Épidémiologie et de Santé Publique (IPLESP UMRS 1136), Paris, France; 3 Univ Paris-Sud, Faculté de Pharmacie, Chatenay-Malabry, France; 4 AP-HP, Hôpital Saint-Antoine, Service de Cardiologie, Paris, France; Ottawa Hospital Research Institute, CANADA

## Abstract

**Background:**

Oral anticoagulation therapy is increasingly used for the prevention and treatment of thromboembolic complications in various clinical situations. Nowadays, education programs for patients treated with anticoagulants constitute an integrated component of their management. However, such programs are usually based on the healthcare providers’ perceptions of what patients should know, rather than on patients’ preferences.

**Objective:**

To investigate patients’ viewpoints on educational needs and preferred modalities of information delivery.

**Methods:**

We conducted an observational study based on a self-administered questionnaire. To explore several profiles of patients, the study was designed for enrolling patients in two settings: during outpatient consultations in a cardiology department (Saint Antoine Hospital, Paris, France) and in community pharmacies throughout France.

**Results:**

Of the 371 patients who completed the questionnaire, 187 (50.4%) were recruited during an outpatient consultation and 184 (49.6%) were recruited in community pharmacies. 84.1% of patients were receiving a vitamin K antagonist and 15.6% a direct oral anticoagulant. Patients ranked 16 of 21 (76.2%) questionnaire items on information about their treatment as important or essential; information on adverse effects of treatment was the highest ranked domain (mean score 2.38, 95% CI 2.30–2.46). Pharmacists (1.69, 1.58–1.80), nurses (1.05, 0.95–1.16), and patient associations (0.36, 0.29–0.44), along with group sessions (0.85, 0.75–0.95), the internet (0.77, 0.67–0.88), and delivery of material at the patient’s home (1.26, 1.14–1.38), were ranked poorly in terms of delivering educational material.

**Conclusion:**

This study revealed substantial discrepancies between patient preferences and current educational programs. These findings should be useful for tailoring future educational programs that are better adapted to patients, with a potential associated enhancement of their effectiveness.

## Introduction

Oral anticoagulation therapy (OAT) is increasingly used for the prevention and treatment of thromboembolic complications in various clinical situations [1]. In 2013, OAT was delivered to 1.5 million patients in France [2]. However, major and minor bleeding is the most common side effect of OAT [3]. The overall risk of major bleeding in warfarin-treated patients is estimated to be 7–8% per year [4], and warfarin is also the most common drug with associated adverse events requiring emergency hospitalizations [5–7]. Data from clinical trials indicate that the risk of major bleeding associated with the newer direct oral anticoagulant drugs remains substantial [8–10].

The interest of patient education as a management component of patients in various health conditions is attested by the availability of several reviews on this topic.[11, 12], Guidelines for managing patients with OAT recommend patient education [13, 14], although recent reviews indicate that the real value of patient education in this population is questionable [15, 16]. In the intention-to-treat analysis of a randomized trial comparing patients who received an educational component and patients managed with usual care, the proportion who experienced major bleeding decreased significantly in the group who had received education (cumulative incidence, 5.6% vs.12%; *P* = 0.0498) [17].

The aims of patient education are to encourage active participation of the patient in his or her healthcare, strengthening his or her ability to manage treatments and symptoms, improve coping strategies, and increase self-care abilities [13]. A broad range of interventions devoted to patient education have been proposed, with various characteristics including patient management by specialized anticoagulation clinics [18, 19], training for self-management or self-monitoring by the patient [20, 21], booklets and videos [22], and multicomponent interventions [23]. Education programs are based primarily on the healthcare providers’ perceptions of what the patients need to know about their health, while few studies in other pathologies have documented patients’ perspectives about education [24–28], and even fewer have described patient preferences regarding the modality of information delivery [24]. Yet, patients’ involvement in healthcare organization and in their treatment has evolved [29, 30]. Involving patients in the design of education programs in terms of what information they need, and what should be included, and how information can optimally be delivered, is likely to provide useful guidance for designing more efficient patient education programs, and, more globally, to enhance healthcare systems oriented towards a patient-centered perspective.

Many patients treated with oral anticoagulants benefit from education regarding this treatment [31, 32]. European guidelines on cardiovascular disease prevention in clinical practice recommend to consider patients’ habits and preferences [14]. However, no study has yet reported the patients’ viewpoint. In order to contribute to the design of more efficient education programs, we undertook a study exploring the point of view of patients treated with OAT on their educational needs: what do patients wish to know, who should deliver the information, and where, when, and how should the information be delivered.

## Materials and Methods

### Study design and setting

This observational study was based on a self-administered anonymous paper questionnaire completed by patients between January and April 2014. To explore needs of several profiles of patients, enrollment was planned in two settings: during outpatient consultations in the cardiology department at Saint Antoine Hospital (Paris); and in community pharmacies throughout France. The community pharmacies recruited in the study were those belonging to a pharmacist network (Humains, Professionnels, et Innovants, http://www.hpisas.com), a nationwide group of 58 community pharmacies certified for their quality of service (ISO 9001). The study was approved by the Ile-de-France VI ethics committee (Comité_de_Protection_des_Personnes/31-14-ID_RCB:2013-A01754-41) and was declared to the National Commission of Informatics (Number 1727672).

In both settings, the participating pharmacy staff received a training note detailing the study and how they should interact with the patient taking the survey. The pharmacist presented and explained the study to all patients being treated with an oral anticoagulant.

### Participants

Patients receiving an OAT for a cardiac indication (for instance, atrial fibrillation, pulmonary embolism, heart valve prosthesis, venous thrombosis…), who were able to read, write, and understand French, and were aged ≥18 years were eligible to participate. Patients treated with OAT after a surgery to replace a hip or knee were excluded. Each patient received an information leaflet about the study. According to French regulations in usual care, patient consent was waived while patient non-opposition was registered.

All patients who fulfilled the above-mentioned inclusion criteria and who were attending an outpatient consultation at the department of cardiology of Saint Antoine Hospital were invited to participate in the study. Those that agreed completed the questionnaire while waiting for their appointment. In the pharmacy setting, all patients who presented to a participating community pharmacy and who fulfilled the study criteria were invited to participate in the study. Patients who agreed completed the questionnaire while at the pharmacy.

### Questionnaire and measurements

The questionnaire was conceived by the authors (a multidisciplinary team in epidemiology, cardiology, pharmacy, with expertise in the domain of patient education) at the light of the literature in the domain of patient educational needs. A pilot investigation involving 20 patients who tested the questionnaire was conducted in January 2014 at the department of cardiology of Saint Antoine Hospital. This pilot study confirmed patient's understanding, feasibility of completion, and resulted in few modifications of the questionnaire proposed to patients in the final survey. The questionnaire included four major sections (see S1 Questionnaire). The first section, on patient demographics, collected data on age, sex, education, and zip code of residence. The second section collected data on treatment characteristics, including anticoagulant name, indication, and time since initiation of treatment. The third section collected data on patient information requirements. This section was composed of 21 items organized into 5 subdomains: daily management of treatment, theoretical knowledge about the treatment and the disease, impact of treatment on lifestyle, treatment adverse effects, and blood tests to monitor the treatment. Respondents were asked to rate each item through a single choice among 5 proposals: *essential*, *important*, *of minor interest*, *unnecessary*, and *I do not wish to answer this question*. Each answer was coded as a score value ranging from 0 to 3 (3 = essential, 2 = important, 1 = of minor interest, and 0 = unnecessary and I do not wish to respond). We calculated a mean score for the 5 subdomains using mean score of each item. For each subdomain, the internal consistency was considered as good (the value of calculated Cronbach's alpha coefficient ranged between 0.82 and 0.91).

The fourth section of the questionnaire investigated 20 items relating to 4 aspects of information delivery: who (appropriateness of given individuals for delivering information), where, when and how to deliver information about treatment. Respondents were asked to rate each item as: *ideal*, *convenient*, n*ot very convenient*, *inappropriate*, or *I do not wish to respond*. Each answer was coded as a score value ranging from 0 to 3 (3 = ideal, 2 = convenient, 1 = not very convenient, 0 = inappropriate or I do not wish to respond).

It is worth noting that a random choice would result in a mean score of 1.5.

### Study size

Several elements were used to determine the study sample size.

First, a pilot investigation, conducted in 2012 at the department of cardiology of Saint Antoine Hospital, indicated that 630 patients on average attended a consultation each month, approximately 20% of whom are treated with an oral anticoagulant. Assuming 50% of these subjects agreed to participate, an estimated 60 patients could be enrolled each month.

Second, according to French national health insurance data, 1.49 million patients were treated with oral anticoagulants in 2013 in France [2], and the College of Pharmacists accounted for 22,461 pharmacies. Therefore, an average of 66 patients per pharmacy is estimated to receive OAT across all indications. A pilot survey conducted in community pharmacies estimated that 50% of the solicited pharmacies would participate, and approximately 10% of the visiting patients on anticoagulant therapy would be eligible and would accept to participate (i.e., about 6 patients per pharmacy).

Therefore, a study conducted in the cardiology department of Saint-Antoine Hospital and at 30 participating community pharmacies over a 3-month period would be expected to enroll approximately 360 patients (180 patients in the hospital and 180 in the pharmacy). This sample size was anticipated to be sufficient to estimate patient preferences with reasonable level of accuracy: for example, the 95% confidence interval (CI) of an item having received one third of favorable opinions would be 28.6% to 38.4%, according to Agresti and Coull.[33]

### Statistical methods

Categorical variables are summarized as counts and percentages and quantitative variables as medians and interquartile ranges, except for scores, which are summarized as means and associated 95% CIs, with the latter obtained by bootstrap. Comparisons on qualitative variables were performed with Fisher’s Exact test while those on quantitative variables were performed with the Wilcoxon-Mann-Whitney test. In order to rank patients’ preferences between several items, nonparametric multiple pair-wise comparisons were performed with the Wilcoxon-Nemenyi-MacDonald-Thompson procedure proposed by Hollander and Wolfe.[34] Multivariable linear regression models were used for investigating factors associated with patients’ preferences.

All analyses were performed through scripts developed in the R software (3.1.0. version). The level of significance was set at *P*<0.05.

Missing data (missing answer for a given item) observed for each of the items related to demographics and treatment characteristics are reported in Table 1. Missing data in the information needs section and the modality of information delivery were interpreted as patients’ lack of interest and were assigned a score value of zero.

**Table 1:** Characteristics of the Survey Respondents (n = 371).

Characteristic			Population (n = 371)	Recruitment location		
Feature	Feature modality	Feature sub-modality		Hospital outpatients (n = 187)	Community pharmacy (n = 184)	*P* value*
Age, median (interquartile range)			71 (62–79.5)	68 (60–77)	74 (64–82)	<0.005
Men, n (%)			214 (57.7)	111 (59.4)	103 (56.0)	>0.2
Education Level, n (%)						>0.2
	Primary school		76 (20.5)	33 (17.6)	43 (23.4)	
	High school		155 (41.8)	78 (41.7)	77 (41.8)	
	College or university		127 (34.2)	71 (38.0)	56 (30.4)	
	Do not wish to answer		13 (3.5)	5 (2.7)	8 (4.3)	
Drug class, n (%)						>0.2
	Vitamin K antagonist		312 (84.1)	154 (82.4)	158 (85.9)	
		Phenindione	261 (70.4)	137 (73.3)	124 (67.4)	
		Coumarin	51 (13.7)	17 (9.1)	34 (18.5)	
	Direct oral anticoagulant		58 (15.6)	33 (17.6)	25 (13.6)	
		Rivaroxaban	34 (9.2)	24 (12.8)	10 (5.4)	
		Dabigatran	23 (6.2)	9 (4.8)	14 (7.6)	
		Apixaban	1 (0.3)	0 (0)	1 (0.5)	
	Missing data		1 (0.3)	0 (0)	1 (0.5)	
Treatment indication, n (%)						<0.05
	Atrial fibrillation		160 (43.1)	90 (48.1)	70 (38.0)	
	Pulmonary embolism		64 (17.3)	38 (20.3)	26 (14.1)	
	Heart valve prosthesis		52 (14.0)	27 (14.4)	25 (13.6)	
	Venous thrombosis		42 (11.3)	11 (5.9)	31 (16.8)	
	Unknown		44 (11.9)	19 (10.2)	25 (13.6)	
	Missing data		9 (2.4)	2 (1.1)	7 (3.8)	
Years since initiation of treatment, n (%)						<0.05
	>5 years		161 (43.4)	69 (36.9)	92 (50.0)	
	1 to 5 years		125 (33.7)	66 (35.3)	59 (32.1)	
	< 1 year		83 (22.4)	51 (27.3)	32 (17.4)	
	Missing data		2 (0.5)	1 (0.5)	1 (0.5)	

*P value for comparison between hospital and community pharmacies.

## Results

### Study population

**Fig 1:**
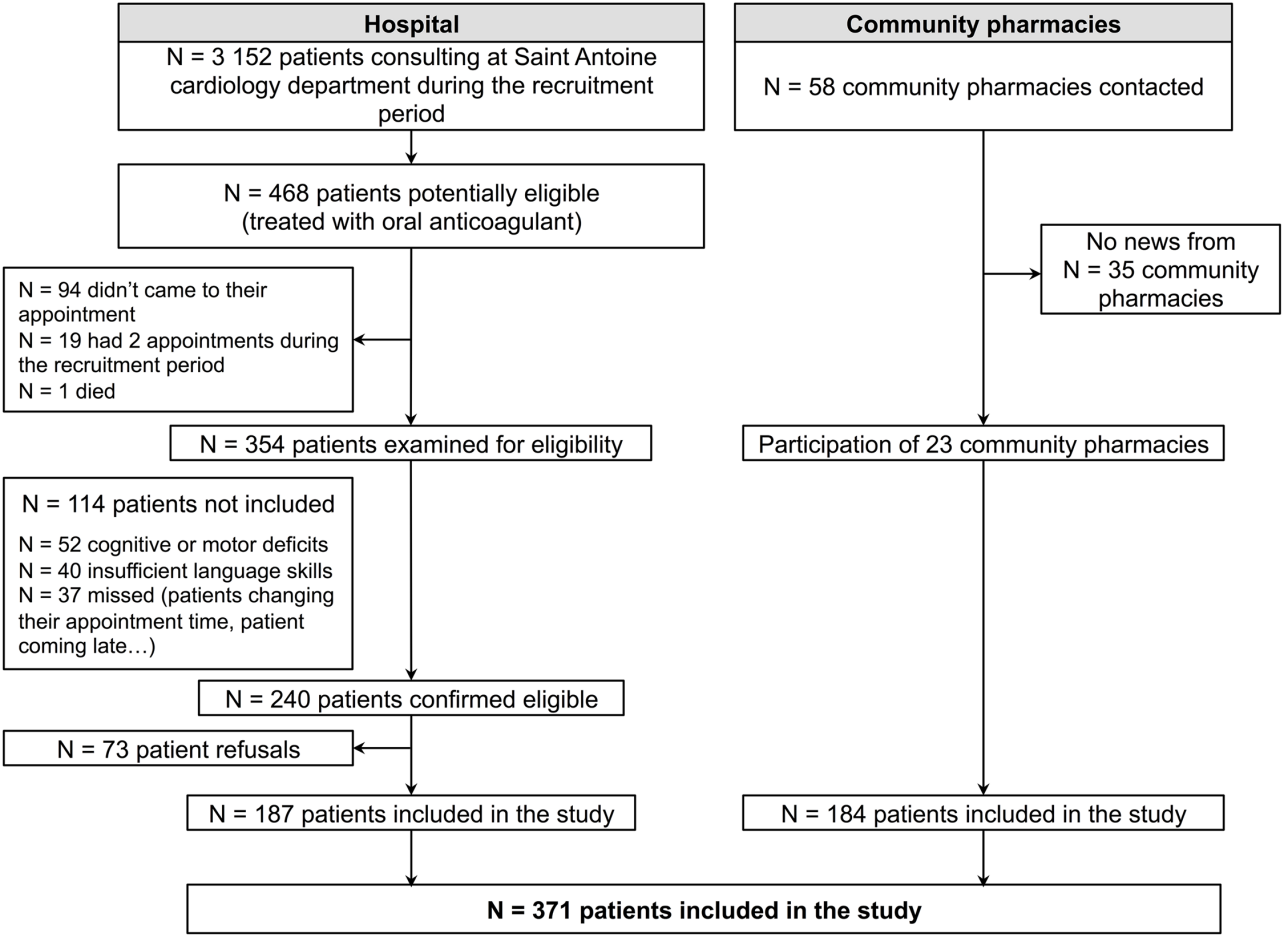
Study flow diagram.

The study flow diagram is shown in Fig 1. Among the 371 patients included in the study, 187 (50.4%) were hospital outpatients recruited in the waiting room at the time of their medical consultation in the Cardiology Department at Saint-Antoine Hospital, and 184 (49.6%) were recruited at the time of their visit in 23 community pharmacies.

The participation rate among hospital outpatients was 77.9% (n = 187/240). For practical reasons, the number of eligible patients in the community pharmacies was not collected. Nevertheless, the estimated number of potentially eligible patients in the 23 participating pharmacies is 1518, according to national data (66 per pharmacy), yielding an estimated participation rate of 12.1% (184/1518).

Patient baseline data are reported in Table 1. The median age of the respondents was 71 (interquartile range 62–79.5) years and 57.7% were men. Overall, 84.1% of patients were receiving a vitamin K antagonist and 15.6% a direct oral anticoagulant. Atrial fibrillation (43.1%) and pulmonary embolism (17.3%) were the most frequent indications. The duration of OAT was ≥5 years in 43.4% of patients.

The distributions of sex, education level, and anticoagulant drug class were not significantly different in hospital outpatients and patients recruited in community pharmacies. However, community pharmacy respondents were older, had a longer treatment history, and had different distribution of indications for treatment, such as a less frequent history of atrial fibrillation and more frequent history of venous thrombosis.

### What: Patients’ preferences on the educational content

The mean scores of the 21 question items concerning education content ranged from 1.68 (95% CI 1.11–2.44) to 2.41 (95% CI 2.32–2.50), and 16 items (76.2%) achieved a mean score >2 (judged to be important or essential). The four top-ranked questions related to information on adverse effects (What side effects are related to anticoagulant therapy, what I should do in the case of an adverse event, are there any interactions with other medicines, what medications can I take without medical advice).

**Fig 2:**
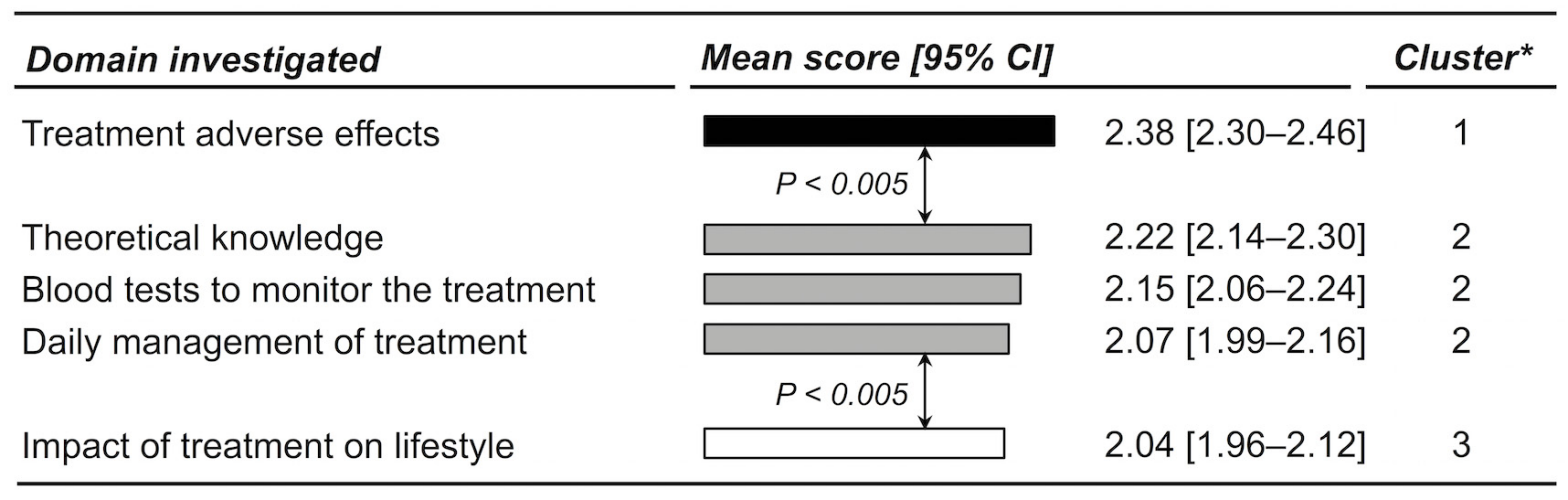
Patients’ perceptions of the importance of several domains related to the content of educational programs. Any two Bars with different shades of grey correspond to subdomains for which the scores were identified as significantly different by the multiple comparison procedure, whereas all bars with a given identical shade of grey correspond to subdomains for which the scores were identified as non-significantly different.

The mean score of the 5 subdomains ranged from 2.04 (95% CI 1.96–2.12) to 2.38 (95% CI 2.30–2.46). The multiple comparison procedure split the 5 subdomains into three statistically different clusters according to the observed scores (Fig 2): information about treatment adverse effects (top-ranked cluster); theoretical knowledge on the treatment and the disease, blood tests to monitor the treatment, and daily management of treatment (second cluster); and treatment impact on lifestyle (third cluster).

### Who, Where, When, and How: patients’ preferred modalities for information delivery

**Fig 3:**
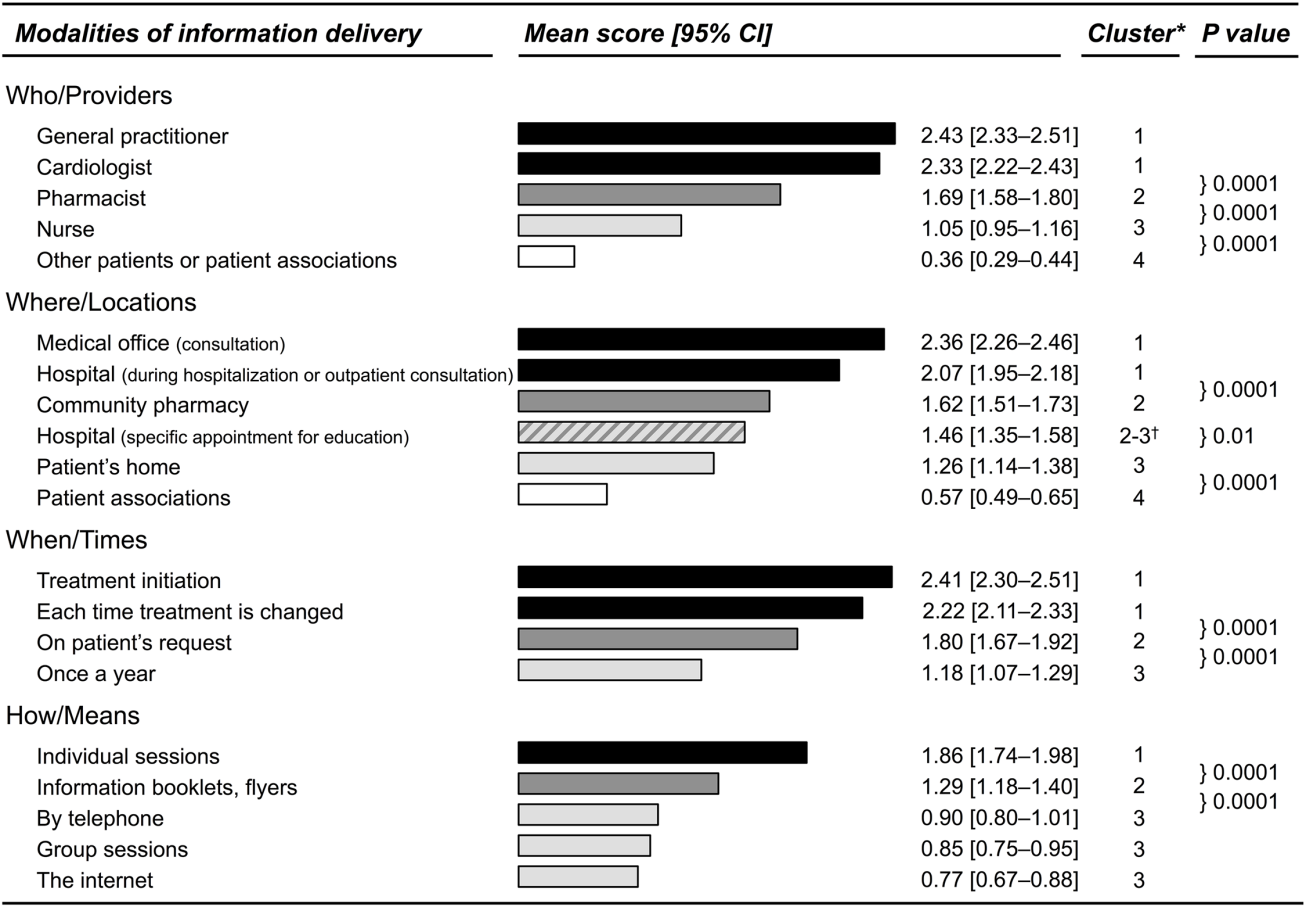
Scores attributed by patients for the explored modalities of information delivery. Within each dimension (What, Where, When, or How), any two bars with different shades of grey correspond to two items for which the scores were identified as significantly different by the multiple comparison procedure, whereas all bars with a given identical shade of grey correspond to items for which the scores were identified as non-significantly different. *Issued from the Wilcoxon-Nemenyi-MacDonald-Thompson test procedure. ^†^Intermediate item: neither significantly different from “community pharmacy” nor from “Patient’s home” (n.b. the P value for the comparison of the two latters groups is 0.01). CI indicates confidence interval.

Patients preferred to receive educational information from their general practitioner (mean score 2.43, 95% CI 2.33–2.51) or their cardiologist (2.33, 2.22–2.43), during a consultation (2.36, 2.26–2.46) or hospitalization (2.07, 1.95–2.18). At initiation of treatment (2.41, 2.30–2.51) and each time a treatment is changed (2.22, 2.11–2.33) were judged to be the most suitable moments for providing information, and patients would prefer to receive the information during individual sessions (1.86, 1.74–1.98) (Fig 3). Conversely, other patients and patient associations (0.36, 0.29–0.44), nurses (1.05, 0.95–1.16), and pharmacists (1.69,1.58–1.80) were not considered to be appropriate entities for delivering information, neither were group sessions (0.85, 0.75–0.95) nor the internet (0.77, 0.67–0.88).

### Exploring the association between score values and patient characteristics

Multivariable linear regression analyses were performed to explore the association between patient preferences (score value) and patient demographics or treatment characteristics (statistically significant associations are shown in S1 Table). Patients recruited in community pharmacies gave a higher score to pharmacists (*P*<0.001) and nurses (*P* = 0.01) and a lower score to cardiologists (*P*<0.001) when compared with hospital outpatients (S1 Fig). Treatment adverse effects, theoretical knowledge about the treatment and the disease, and impact of treatment on lifestyle were three subdomains judged significantly more important by younger patients. In addition, group sessions, booklets, and the internet as a means of information delivery were judged more favorably by young patients. Patients treated with vitamin K antagonists gave a significantly higher score (*P*<0.001) to the subdomain of blood tests to monitor the treatment when compared to patients treated with a direct oral anticoagulant (S2 Fig). The scores attributed to information domains were not significantly associated with treatment duration.

## Discussion and Conclusion

### Discussion

Based on the opinions of 371 respondents who are being treated with oral anticoagulants, this study reports patients’ preferences in terms of educational programs about their disease and its treatment. In each of the dimensions explored (information wished and preferences of patients for the modalities of information delivery: who is the preferred entity for delivering information, and where, when, and how information should be delivered) patients ranked propositions and analysis revealed some items significantly more appreciated than others. Patients ranked 76.2% of the information items to be either important or essential, with advice on the adverse effects of treatment being ranked the highest. In terms of preferences for delivering educational materials, patients gave a higher score to physicians along with individual sessions; medical office and hospital at the beginning of the treatment or when the treatment is changed were judged the most appropriate places and times to receive education session. Conversely, pharmacists, nurses, and patient associations ranked poorly, along with group sessions, the internet, and delivery of information at the patient’s home. The study results should be helpful for the design of future educational programs, more closely aligned to patients’ preferences. Taking into account patients’ preferences in the design of educational programs might enhance their effectiveness.

This study has some limitations, the first of which concerns the study population, with potential selection bias. Only one hospital department was involved in patient recruitment and the 30 community pharmacies were not sampled at random. However, the hospital outpatients enrolled in this study are likely to be similar to outpatients treated with oral anticoagulants in other hospitals, and patients from community pharmacies were recruited in 19 of the 22 regions in France. In addition, the balanced recruitment in hospital and community pharmacies in this study is likely to result in a participant sample with potential complementary patterns. Furthermore, the planned sample size resulted in satisfactory CIs for the estimates. A second limitation concerns the coverage of items in the questionnaire: Patients’ preferences about items that were not included, such as video material [35], remain unknown. Nevertheless, the questionnaire allowed the investigation of patients’ preferences on major aspects of educational programs on anticoagulant therapy. A third limitation concerns lack of knowledge about background information regarding patients‘ current anticoagulant management, It would be interesting to assess whereas the need for information was related to actual knowledge of the patients.

This study has also many strengths. It highlighted patients’ major concerns about treatment adverse effects. A study on warfarin therapy [36] and studies on other diseases [24, 28, 37] report similar patients’ perceptions of treatment adverse effects. Therefore, being well informed about potential adverse effects of treatment appears to be a major concern for patients, whatever their underlying disease and treatment. Importantly, our study revealed substantial discrepancies between patients’ preferences and usual educational program practices, especially in terms of the modalities for information delivery. Whereas participants in our study ranked pharmacists, nurses, and other patients or patient groups poorly in terms of delivering information, a systematic review on patient education strategies [38] identified 11 of 32 articles describing individuals involved in the educational strategy, with nurses, pharmacists, and physicians, respectively involved in 6, 4, and 3 articles. In addition, the French National Health Insurance decided in 2013 to finance pharmacists for delivering individual sessions to patients treated with vitamin K antagonists. Moreover, peer support interventions are increasingly being implemented and are believed by some to be a promising approach to help patients manage their chronic conditions [39, 40]. Indeed, our results suggest that patients’ expectations contrast deeply with current or planned educational programs. The lower ranking of pharmacists and nurses might be explained by patients’ relatively low exposure to these health care providers. Since an increasing involvement of pharmacists in this type of counselling is planned in France, a reassessment of patients' opinions on pharmacists after the corresponding increased exposure would be interesting. Discrepancies also exist for other aspects of information delivery. In our study, group sessions received a low rank, whereas the systematic review by Wofford et al [38] indicated that 9 of 13 articles described education programs involving group sessions. Our study also revealed that the internet ranked poorly as a method for providing information. In contrast, the development of a wide range of health information websites and new eHealth applications promise to increase patient access to relevant health information [41]. This discrepancy might be explained by the age of the study participants, which reflects the current culture gap between elderly people and the internet [42, 43].

### Conclusion

In conclusion, this study reveals important discrepancies between patients’ preferences and current educational program practices. These results may be helpful for designing education programs that are more closely aligned to patients’ preferences. Tailoring educational programs that take into account patients’ opinions may enhance their effectiveness.

## Supporting Information

S1 Dataset Individual data about patients’ characteristics and answers to the questionnaire used in the study. (XLSX)

S1 Fig Influence of place of recruitment (hospital outpatient clinic or community pharmacy) on respondents’ scores. CI indicates confidence interval.
(TIFF)

S2 Fig Information domain scores according to treatment class. CI indicates confidence interval; DOA, direct oral anticoagulants; and VKA, vitamin K antagonists.
(TIFF)

S1 Questionnaire Survey questionnaire. (DOCX)

S1 Table Significant Associations* (P≤0.05) Between Patient Demographic or Treatment Characteristics and Patient Scores *. Multivariable linear regression models were used for investigating factors associated with patients’ preferences. All analyses were performed with the R software (version 3.1.0). **OAC indicates oral anticoagulant. *Hospital outpatient consultation or community pharmacy.
(DOCX)
