# Supplementary material for: Discrepancies between Patients’ Preferences and Educational Programs on Oral Anticoagulant Therapy: A Survey in Community Pharmacies and Hospital Consultations
Source: PLoS One. 2016 Jan 14;11(1):e0146927. doi: 10.1371/journal.pone.0146927 (PMC4713069; doi:10.1371/journal.pone.0146927)
Supplement: S1 Questionnaire — (DOCX) [file pone.0146927.s004.docx]

**ORAL ANTICOAGULANT QUESTIONNAIRE**

**Administrative information**

1. What is today’s date?

2. What is your date of birth?

3. What is your gender?

□ male □ female

4. What is your zip code?

5. What is the highest level of education you have completed?

□ Primary education □ High school □ College or university □ I do not wish to answer

**Information about your anticoagulant therapy**

6. What is your current oral anticoagulant treatment?

□ Previscan® (fluindione) □ Pradaxa® (dabigatran)

□ Sintrom® or Minisintrom® (acenocoumarol) □ Xarelto® (rivaroxaban)

□ Coumadine® (warfarin) □ Eliquis® (apixaban)

7. How long ago did you start to take oral anticoagulation therapy? (if you have received anticoagulant treatment several times, please use the date of the first treatment)

□ Less than 3 months □ Over 5 years

□ Between 3 months and 1 year □ I do not remember

□ Between 1 and 5 years

8. What is the indication (reason) for your anticoagulant therapy?

□ Atrial fibrillation (arrhythmia) □ Deep vein thrombosis (phlebitis)

□ Heart valve prosthesis □ I don’t know

□ Pulmonary embolism □ Other (please specify): …………………………..

9. Considering your current knowledge, do you wish to have more information about your anticoagulant therapy? Tick the appropriate answer.

□ I want more information because I do not have any information

□ I want more information because the information I have is insufficient

□ I want more information because the information I have is unsuitable

□ I do not want more information because the information I received was sufficient and appropriate

□ I do not want more information because my doctor manages my treatment

□ I do not want more information because it scares me to know too much about my treatment and my disease

**WHAT INFORMATION WOULD YOU LIKE TO RECEIVE ON YOUR TREATMENT?**

We would like to know how important or not you consider the information given to you about your anticoagulation therapy.

For each question, whether you have received the information or not, state your opinion by ticking a possible answer:

- Essential information
- Important information
- Information of minor interest
- Unnecessary information

The information has been grouped into five themes (questions 10–14) :

- Daily management of treatment
- Theoretical knowledge
- Impact of treatment on lifestyle
- Treatment adverse effects
- Blood tests to monitor the treatment

10. DAILY MANAGEMENT OF TREATMENT

How important do you think the following information is (whether or not you have received it)?

|  | Essential  information | Important  information | Information of minor interest | Unnecessary  information | I do not wish to respond |
| --- | --- | --- | --- | --- | --- |
| How to take my anticoagulant medication | □ | □ | □ | □ | □ |
| What to do if I miss a dose | □ | □ | □ | □ | □ |
| How to avoid running out of medication | □ | □ | □ | □ | □ |
| If you are taking Previscan® (fluindione): how to manage splitting tablets | □ | □ | □ | □ | □ |

11. THEORETICAL KNOWLEDGE ABOUT THE TREATMENT AND THE DISEASE

How important do you think the following information is (whether or not you have received it)?

|  | Essential  information | Important  information | Information of minor interest | Unnecessary  information | I do not wish to respond |
| --- | --- | --- | --- | --- | --- |
| Why my disease requires anticoagulation treatment | □ | □ | □ | □ | □ |
| How the disease will progress | □ | □ | □ | □ | □ |
| How long my anticoagulation therapy will last | □ | □ | □ | □ | □ |
| What is the purpose of my anticoagulation treatment | □ | □ | □ | □ | □ |
| Why it is important to take my anticoagulant therapy every day | □ | □ | □ | □ | □ |

12. IMPACT OF TREATMENT ON LIFESTYLE

How important do you think the following information is (whether or not you have received it)?

|  | Essential  information | Important  information | Information of minor interest | Unnecessary  information | I do not wish to respond |
| --- | --- | --- | --- | --- | --- |
| Whether I need to change my diet | □ | □ | □ | □ | □ |
| What types of exercise I can do | □ | □ | □ | □ | □ |
| What I have to do if I want to travel | □ | □ | □ | □ | □ |
| Who I should tell about my anticoagulation treatment (doctor, pharmacist, family, etc.) | □ | □ | □ | □ | □ |
| If relevant: What to do if I want to become pregnant | □ | □ | □ | □ | □ |

13. TREATMENT ADVERSE EFFECTS

How important do you think the following information is (whether or not you have received it)?

|  | Essential  information | Important  information | Information of minor interest | Unnecessary  information | I do not wish to respond |
| --- | --- | --- | --- | --- | --- |
| What side effects are related to anticoagulant therapy | □ | □ | □ | □ | □ |
| What I should do in the case of an adverse event | □ | □ | □ | □ | □ |
| Are there any interactions with other medicines | □ | □ | □ | □ | □ |
| What medications can I take without medical advice | □ | □ | □ | □ | □ |
| How can I identify dangerous situations | □ | □ | □ | □ | □ |

14. BLOOD TESTS TO MONITOR TREATMENT

How important do you think the following information is (whether or not you have received it)?

|  | Essential  information | Important  information | Information of minor interest | Unnecessary  information | I do not wish to respond |
| --- | --- | --- | --- | --- | --- |
| Why I need to undergo blood tests | □ | □ | □ | □ | □ |
| How to interpret the results of blood tests | □ | □ | □ | □ | □ |
| How often I have to have blood tests | □ | □ | □ | □ | □ |

**HOW WOULD YOU LIKE TO RECEIVE INFORMATION ON YOUR TREATMENT?**

We would like to know your opinion on how we should deliver information about your anticoagulant therapy: who should provide it, where, when and how?

For each proposal, state your opinion by ticking a possible answer:

- Ideal
- Convenient
- Not very convenient
- Inappropriate
- I do not wish to respond

15. What do you think about the following people for delivering information about your anticoagulant treatment? (Tick one answer per line)

|  | Ideal | Convenient | Not very convenient | Inappropriate | I do not wish to respond |
| --- | --- | --- | --- | --- | --- |
| General practitioner | □ | □ | □ | □ | □ |
| Cardiologist | □ | □ | □ | □ | □ |
| Pharmacist | □ | □ | □ | □ | □ |
| Nurse | □ | □ | □ | □ | □ |
| Other patients | □ | □ | □ | □ | □ |

16. What do you think about the following locations for delivering information about your anticoagulant treatment? (Tick one answer per line)

|  | Ideal | Convenient | Not very convenient | Inappropriate | I do not wish to respond |
| --- | --- | --- | --- | --- | --- |
| At hospital, during hospitalization | □ | □ | □ | □ | □ |
| At hospital, not during hospitalization | □ | □ | □ | □ | □ |
| At a consultation | □ | □ | □ | □ | □ |
| At a pharmacy | □ | □ | □ | □ | □ |
| At a patient organization | □ | □ | □ | □ | □ |
| At home | □ | □ | □ | □ | □ |

17. What do you think about the following times for delivering information about your anticoagulant treatment? (Tick one answer per line)

|  | Ideal | Convenient | Not very convenient | Inappropriate | I do not wish to respond |
| --- | --- | --- | --- | --- | --- |
| At treatment initiation | □ | □ | □ | □ | □ |
| Each time treatment is changed | □ | □ | □ | □ | □ |
| Once a year | □ | □ | □ | □ | □ |
| At my request | □ | □ | □ | □ | □ |

18. What do you think about the following means for delivering information about your anticoagulant treatment? (Tick one answer per line)

|  | Ideal | Convenient | Not very convenient | Inappropriate | I do not wish to respond |
| --- | --- | --- | --- | --- | --- |
| Individual sessions | □ | □ | □ | □ | □ |
| Group sessions | □ | □ | □ | □ | □ |
| By telephone | □ | □ | □ | □ | □ |
| Information booklets, flyers, etc | □ | □ | □ | □ | □ |
| Specialized sites on the internet | □ | □ | □ | □ | □ |

19. Do you have any comments?
