# Supplementary material for: Discrepancies between Patients’ Preferences and Educational Programs on Oral Anticoagulant Therapy: A Survey in Community Pharmacies and Hospital Consultations
Source: PLoS One. 2016 Jan 14;11(1):e0146927. doi: 10.1371/journal.pone.0146927 (PMC4713069; doi:10.1371/journal.pone.0146927)
Supplement: S1 Table — Multivariable linear regression models were used for investigating factors associated with patients’ preferences. All analyses were performed with the R software (version 3.1.0). **OAC indicates oral anticoagulant. *Hospital outpatient consultation or community pharmacy. (DOCX) [file pone.0146927.s005.docx]

**eTable 1. Significant Associations* (*P*≤0.05) Between Patient Demographic or Treatment Characteristics and Patient Scores**

| Questionnaire sections | Population characteristics | | | | | | |
| --- | --- | --- | --- | --- | --- | --- | --- |
|  | Recruitment place** | Age | Sex | Education | Drug class | Treatment duration | Indication for OAC |
| WHAT: DOMAIN INVESTIGATED |  |  |  |  |  |  |  |
| Treatment adverse effects |  |  |  |  |  |  | 0.002 |
| Theoretical knowledge |  |  |  |  |  |  |  |
| Blood tests to monitor the treatment |  |  |  |  | 0.0006 |  |  |
| Daily management of treatment | 0.025 |  |  |  |  |  |  |
| Impact of treatment on lifestyle |  | 0.008 |  |  |  |  |  |
| MODALITY OF INFORMATION DELIVERY | | | | | | | |
| WHO |  |  |  |  |  |  |  |
| General practitioner |  |  |  |  | 0.05 | 0.05 |  |
| Cardiologist | 0.001 |  |  |  |  |  | 0.01 |
| Pharmacist | 0.001 | 0.05 | 0.05 |  |  |  |  |
| Nurse | 0.01 | 0.01 |  | 0.05 |  |  |  |
| Other patients or patient associations |  |  |  |  |  |  |  |
| WHERE |  |  |  |  |  |  |  |
| Medical office (consultation) |  |  |  |  |  |  |  |
| Hospital (during hospitalization or outpatient consultation) |  | 0.05 |  |  |  |  |  |
| Community pharmacy | 0.001 | 0.001 |  |  |  |  |  |
| Hospital (specific appointment for education) | 0.001 | 0.01 |  |  |  |  |  |
| Patient’s home |  |  |  |  |  |  |  |
| Patient associations |  | 0.01 |  |  |  |  |  |
| WHEN |  |  |  |  |  |  |  |
| On initiation of treatment | 0.01 | 0.01 |  |  |  |  |  |
| Each time treatment is changed |  |  |  |  |  |  |  |
| Once a year |  |  |  |  |  |  |  |
| On patient’s request |  | 0.05 |  |  | 0.05 |  |  |
| HOW |  |  |  |  |  |  |  |
| Individual sessions |  |  |  |  |  | 0.05 |  |
| Information booklets, flyers |  | 0.05 |  |  |  |  |  |
| By telephone |  | 0.05 |  |  |  |  |  |
| Group sessions |  | 0.01 |  |  |  |  |  |
| The internet |  | 0.001 | 0.05 |  |  |  |  |

* Multivariable linear regression models were used for investigating factors associated with patients’ preferences.

All analyses were performed with the R software (version 3.1.0).

**OAC indicates oral anticoagulant. *Hospital outpatient consultation or community pharmacy
